# Pathology and oncology in Africa: education and training for the future in cancer research– East African Regional Meeting

**DOI:** 10.1186/s13027-015-0044-7

**Published:** 2015-12-17

**Authors:** D. C. Stefan, N. Masalu, L. Ngendahayo, D. Amadori, M. Botteghi, M. Mendy, N. A. Othieno-Aabinya, T. Ngoma, F. Asirwa, O. Balogun, W. Ngwa, E. Vuhahula, A Adesina

**Affiliations:** South African Medical Research Council, Parrow, Cape Town, 7550 South Africa; Department of Oncology, Bugando Medical Centre , Mwanza, Tanzania; Department of Pathology, University Teaching Hospital of Kamenge, University of Burundi, Bujumbura, Burundi; Institute for cancer research and Care, IRST, Meldola, Italy; Department of Clinical and Molecular Sciences Experimental Pathology Research Group, Vittorio Tison Association, Italy Marche University, Ancona, Italy; Laboratory Services and Biobank Group, International Agency for Research on Cancer, Lyon, France; University of Nairobi, Nairobi, Kenya; Muhimbili University of Health and Allied Sciences, Dar es Salaam, Tanzania; Academic Model Providing Access to Healthcare (AMPATH), Eldoret, Kenya; New York Presbyterian/Weill Cornell Medical Center, New York, USA; Brigham and Women’s Hospital, Dana Farber Cancer Institute, Boston, USA; Department of Pathology, Texas Children’s Hospital, Baylor College of Medicine, Houston, TX USA

**Keywords:** Pathology, Oncology, Eastern Africa, Education, Research

## Abstract

According to the World Health Organisation (WHO), deaths from non-communicable diseases (NCDs) will increase globally, with the largest increase being on the African continent. On our continent, projections have indicated that deaths from NCDs will exceed all combined communicable, maternal, perinatal and nutritional diseases as the most common causes of death by 2030. Hence, the importance of a functional and improved pathology system in the diagnosis of cancer cannot be debated.

Recently, the African Organization for Research and Training in Cancer (AORTIC) organised its East African regional meeting in Mwanza, Tanzania on 25–26 June 2015, with the focus being ‘Pathology and oncology: Education and training for the future in cancer research’. The main themes of the workshop were around improving cancer care and the role of twinning in Eastern Africa, in particular the Mwanza cancer project, telepathology, e-health and biobanking. The outcomes of a 2 day strategic meeting were developing an efficient and effective plan to guide the improvement in pathology training and cancer research in Africa.

## Introduction

Africa is a continent of opportunities for growth. It is a continent undergoing a rapid economic transformation, which, however, will result in an increase of non-communicable diseases (NCDs). Estimates suggest that the annual number of new cases over the next 5 years will grow to above 1 million. Predicted deaths from NCDs over the next 10 years will also increase globally by 17 %, the greatest increase being in Africa (27 % or 28 million deaths) [1].

The importance of pathology in correct diagnostics and further adequate cancer treatments cannot be emphasized enough. There are currently still a high number of African countries in which pathology services are struggling with a limited number of pathologists, inadequate infrastructure and with severely restricted budgets from the government. At the same time, pathology is unarguably the backbone of the success of the cancer care.

In June 2015, AORTIC (African Organization for Research and Training in Cancer) organised its East African regional meeting in Mwanza, Tanzania on 25–26 June 2015, with the focus being ‘Pathology and oncology: Education and training for the future in cancer research’. This meeting is the first in a series of regional meetings expected to follow the African Pathologist’ Summit organised by Aortic in Dakar, Senegal in 2013.

The emphasis of the meeting was on explorating regional resources, including professional and technical manpower that will be critical for improving pathology diagnostics, clinical oncology, research, as well as education and training in the region. In the past, there has been a lot of talk about the role of African pathology in the context of clinical oncology, often with very limited follow up and action.

The meeting was a strategic meeting attended by 26 participants coming from 21 institutions and 8 countries.

This meeting produced a list of initiatives that resulted in a joint collaboration within East Africa, integrating pathology, clinical oncology, education and training, and research, bringing together scientists, governments, diaspora and international organizations.

### The role of pathology in cancer care

After a short introduction by the vice-president of Aortic Eastern Africa, Louis Ngendahayo, pathologist from Burundi, a welcome address by the local organiser Nestory Masalu, and an opening address by the secretary treasurer elect Nicholas Othieno-Abinya, the first talk was given by the chair of the Aortic pathology group, Adesina Adenkule. The chair talked about the role of pathology in cancer care and interacted with the audience, raising the question of meeting the needs of this issue in Africa.

The challenges facing pathology diagnostics, education and oncology research in Africa, and specifically in East Africa, are multiple and daunting. They include, inter alia, (i) the lack of or inadequate infrastructure, (ii) inadequate personnel: both pathologists and technical personnel, (iii) inadequacy of opportunities for professional education or training, (iv) ‘brain drain’ following many years of political military taking over with mismanagement of the health-care services and (v) a lack of or insufficient funding for basic laboratory materials such as reagents.

While we acknowledge these challenges, many of which are not going to go away in the foreseeable future, custodians of African and specifically East African pathology and clinical oncology must begin to ask new and pragmatic questions, for example: How can we deliver a better and more acceptable quality of services within the limits of current resources? The ‘game plan’ must address the following, immediately and in a sustained way. It must:

- explore avenues for updating the knowledge base of practicing pathologists in a sustainable way, addressing the processes for enhancing the quality of training of current pathology trainees and technical staff
- explore the need for continuous quality improvement and quality assurance
- develop training in appropriate new technologies when relevant to the level of practice
- address the need for advocacy to both private funding agencies (local and international) and government/ministries of health.

In executing this game plan, urgent attention must be given to database development to provide information on current status and ongoing activities as we move towards measuring success versus failure. In addition, there must be an emphasis on education and training. It is crucial to define short-term (immediate deliverables) and long-term educational goals. Pathology education will most likely yield early dividends in improved patient care and outcomes. However, long-term dividends will only come with the development of local advocacy and subsequent improvement of infrastructure.

### ‘Cancer in Africa: what needs to be done?’

The keynote address was given by Twalib Ngoma, previous Aortic president, who underlined that cancer is often not seen as a priority in many African countries. Out of the 6 % of the gross national expenditure that is spent on health care in Tanzania, less than 0.1 % of available resources for health services are allocated for such services in the oncology field, even though 8–10 % of the disease burden in Africa is due to cancer.

The present situation in Africa is dominated by a low cancer awareness, inexistent or inefficient cancer prevention, overburdened health systems, and inadequately funded and structurally challenged health systems to meet the expected doubling of the number of cancer patients in the next 15–20 years.

A situation like this needs to be remedied urgently by changing the mind-set of Africans about cancer, understanding the magnitude of the problem of cancer in Africa and formulating realistic implementable National Cancer Control Plans (NCCP).

The availability of cancer registries, which have accurate information and data on the national and local situation, is crucial for the planning, implementing and evaluating the NCCP.

The lecture was followed by a discussion group led by three panellists who represented local pathologists from Tanzania, Uganda and Kenya. The panellists led a discussion on defining practice pathology goals in the East African resource-poor environment to a high standard.

The second part of the first day was dedicated to the role of telemedicine, telepathology, e-health for clinical oncology and a practical demonstration of digital pathology. The demonstration took place at Bugando Medical Centre Mwanza, Tanzania.

### Cancer control project and intervention model in the Mwanza region

Until 2009 in Tanzania, an African country with a population of over 50 million inhabitants, only one hospital (Ocean Road Cancer Institute in Dar El Salam) offered systematic cancer treatment.

The Mwanza Cancer Project, the idea of the Vittorio Tison Association in Italy, in collaboration with the government of Tanzania gave rise to the second centre situated in the north-west part of the country. The evolution of this project was co-presented by Dino Amadori and Nestory Masalu This was a comprehensive cancer centre dedicated to cancer care and control, covering all the issues related to cancer, from community awareness and education through early detection and treatment, to palliative care and surgery, based on a structured cancer programme.

The cancer control project is structured into four main intervention areas: prevention and early diagnosis, assistance and therapy, education, and research.

#### Anatomic pathology laboratory

The establishment of the histopathology laboratory started in 2000, with the Italians sponsoring the training of a pathologist, a medical oncologist and four oncology nurses. Soon after that, Bugando Medical Centre became a specialised hospital in north-west Tanzania. The hospital has 850-bed capacity for a catchment population of 16 million, equivalent to one-third of the population of Tanzania.

The histopathology laboratory, which currently has two pathologists, has the capacity to perform 4 000 histological analyses and 1 000 cytologies per year. The technical diagnostic activity of the laboratory is supported by a local professional team, and by Italian volunteer pathologists and technicians that periodically work in this structure. The Association continues to offer support for both diagnostic activity management and new technology development.

#### Medical oncology unit

Shortly after the opening of the laboratory, an oncology department was created, initially receiving a total of 40 beds. In the past 4 years, the department has experienced a 10-fold growth in the number of patients, which has increased from 300 new cases in 2010 to more than 3 000 in 2014.

As patients tend to present with the advanced disease, there is a great need for a palliation centre. This specialized service has been integrated into the existing home-based care programme for patients with HIV/AIDS. Annually, approximately 300 patients with advanced disease make use of the facility’s services, while double this number are under community-based care.

In the last few years, the focus was on prevention with the introduction of a screening programme which has been initiated for breast and cervical cancer with more than 4 000 women enrolled in the last 3 years.

#### Medicines supply

The Association, in collaboration with IRST, is responsible for the supply of drugs which are unavailable in the country, in order to guarantee the best therapeutic quality and continuity of treatment to cancer patients.

#### Radiotherapy

The Association donated a linear accelerator. The accomplishment of establishing a high-level radiotherapy facility in a geographical area that is completely underserved in this field, represents a fundamental improvement of the quality of cancer therapy in that country.

#### Education

The Association supports permanent educational and professional training programmes for the Bugando Medical Center health-care assistants through residential internships in oncological structures, specific educational events, academic specialisation courses and 1st and 2nd level master’s degrees.

#### Research

Research is a strategic area in order to provide high level assistance in oncology. On the initiative of Vittorio Tison Association, in August 2012, AFRICOG (African Italian Cooperative Oncology Group) was founded with the purpose of developing clinical and epidemiological research activities among the sub-Saharan population.

#### X Vittorio Tison Association “Culture & Solidarity” ONLUS

Founded in 1999 the association aims to promote cultural and scientific initiatives, both in Italy and abroad, on issues of human solidarity, in particular in the field of health and social care in oncology.

### Telepathology and application in pathology diagnostics

The next presentation was given by Matteo Botteghi who described telepathology, and the basics and application in pathology diagnostics.

He underlined that the incidence of pathologies like tumors and infections in developing countries is dramatically increasing, currently representing a significant public health burden. The ability to provide early diagnosis, treatments, and follow-up care has a large impact on care efficacy and patient survival.

Telemedicine is defined as the delivery of health-care services in areas where distance is a critical factor, for all health-care professionals by using information and communication technologies (ICT) to exchange valid information for diagnosis, treatment and prevention of disease and injuries, research and evaluation, and for the continuing education of health-care providers, all in the interests of advancing the health of individuals and their communities.

Although telemedicine implementation largely remains a privilege of developed countries with more economic resources, it could be of great use and efficacy in developing countries that lack appropriate health-care facilities by encouraging good health-care practices.

The World Wide Web and ICT play a pivotal role in telemedicine diffusion. ‘Digital Divide’ is a term that describes the economic and social inequality of access, use and knowledge of the Internet and ICT. Telemedicine has the potential to be used in many services and applications such as Voice over IP telephony, web conferencing, teleconsulting, medical records software, remote digital imaging, e-learning, video broadcasting, robotic remotized systems, remote surgery, home-care solutions, e-Hospital, and several others. Service and equipment are divided into categories: Network Critical Physical Infrastructures (NCPI), network connectivity (Internet and other networks), network devices, ICT applications, electromedical devices, and private and public hosted services.

In low-income countries, cost and the availability of Internet connections are limiting factors to the diffusion of telemedicine. In sub-Saharan Africa, there are a limited number of low-cost telemedicine solutions available, for example, using long wireless links to carry clinical data and remote counseling.

In the field of telepathology, the Italian NGO, Pathologists Beyond Borders, has 20 years of experience, in several African countries, of implementing satellite data transfer in very poor and disadvantaged situations, in order to perform remote diagnosis. The large number of pending open issues tells us that we now need a more adequate and modern approach to deal with telemedicine requirements.

Matteo’s presentation also described the telemedicine project between Istituto Scientifico Romagnolo per lo Studio e la Cura dei Tumori (IRST) in Meldola (Italy) (IRST), Italy, and the Bugando Medical Centre Mwanza, Tanzania, initiated in 2012: the ‘Share & Mee’ telemedicine project. Share & Meet includes a novel telematics platform, which is oriented to oncology and its related branches. The project is developed with cooperative programmes, including training medical staff, remote pathology diagnosis and other e-oncology applications.

### The value of e-health in clinical oncology

One of the panellists, Wil Ngwa (part of the African diaspora), presented the value that e- health has for clinical oncology. According to the World Health Organization, e-health is defined “as the transfer of health resources and health care by electronic means. It involves delivery of health information for health professionals and consumers via information and communication technologies (ICTs) e.g. the Internet and mobile phones. It also includes using the power of ICTs to improve public health services, e.g. through the education and training of health workers and related research. ”

There is a growing consensus that ICTs have tremendous potential to catalyse global health collaborations. Advanced ICTs can be employed to elide space-time limitations to leverage a major recent upsurge in global health interest into greater space-time flexible collaborative action against cancer. There are currently a number of emerging models for e-health collaborations in clinical oncology that harness the power of ICTs. The Global Health Catalyst (GHC) programme at the Dana Farber/Harvard Cancer Center (DF/HCC) represents one such model aimed at making it easier for anyone interested in e-health to participate, promoting knowledge-sharing, and helping to scale-up or expand successful clinical oncology e-health models in cancer care, research and education. There is a plan to roll out a number of initiatives that will directly benefit e-health for clinical oncology in Africa.

More specifically, in clinical oncology, some e-health models include tumor boards, remote consultation support via phone or the Internet, remote treatment planning and quality assurance. These are all models that can be scaled or extended to benefit many African country institutions in a lower-cost approach to help eliminate prevailing disparities in clinical oncology. Meanwhile, under education, online learning represents the future. The African Organization for Research and Training in Cancer (AORTIC) is already working to take advantage of ICTs for education and training. As part of the roadmap for cancer control in Africa, AORTIC’s Education and Training (E&T) committee is building virtual infrastructure and resources that will benefit e-health in E&T. This will follow a short survey to better assess the existing workforce, educational pathways, and needs and access to ICTs. Results will be presented at the AORTIC 2015 conference in Morocco. An AORTIC virtual education platform will facilitate access to continuous education modules and other programmes that could significantly help build capacity for clinical oncology in Africa. If well-coordinated, the growing use of ICTS via these models could significantly impact e-health for clinical oncology in Africa, and AORTIC members are particularly encouraged to take advantage and let others know about these emerging opportunities.

### An overview of cancer situation in Africa

The second day started with an overview of the cancer situation in Africa, diagnostic and treatment facilities, as well as an overview of paediatric oncology in Africa and cost-effective treatments. The presentation was given by Cristina Stefan, AORTIC vice-president, Southern Africa. She described how starting with the end in mind allows us to develop plans for each East African country, and develop strategies for implementation and common principles in controlling cancer on the continent.

In Africa, projections have indicated that deaths from NCDs will exceed all combined communicable, maternal, perinatal and nutritional diseases as the most common causes of death by 2030.

There are 19 million new cases of cancer predicted by 2025, with a remarkable increase in low- to middle- income countries. The growth of cancer incidence on the African continent is considerable when viewed in the context of a century of a double or triple burden disease, with cancer now representing a real developmental problem. The survival of people with cancer in Africa is far behind the figures in high-income countries. For example, the 5-year survival ratio of women with breast cancer [2] in Europe is 82 %, in Uganda it is 46 %, 39 % in Algeria and in Gambia it is only 12 %.

Africa is experiencing a unique health trajectory with huge differentials between African countries on basic spend budget for health.

Africa’s burden is uncertain, but whatever the ‘real’ numbers are, cancer cases and other NCDs are going to be a major burden. Population increase, increased life span and modifiable risk factor exposure are all contributing factors. It is not yet known how HIV/AIDS will change the burden of NCDs. In this context, prevention remains essential even if it is translated in very high costs.

Africa is moving in the right direction to acknowledge the present impact and future burden of cancer on the continent. The correct strategies for faster implementation are required, as are sustainable and viable solutions.

At the end of her presentation, Dr Stefan proposed some innovative ideas such as a case for a global cancer fund, expanding the Bugando model to eastern Africa, telepathology and a consideration for an African pathology school.

### Strategies for improving oncologic care in East Africa

The second presenter of the day was Nicholas Othieno-Abinya, the secretary-elect of AORTIC.

Africa is the second largest continent, with a population of 1.1 billion people, second only to Asia. It arguably has the highest concentration of natural resources, but it is also the most resource-challenged continent. About 800 000 new cancer cases occur in 2012 and the figure is to be closer to 1.4 million annually within the next two decades [3]. Breast cancer and cervical cancers are on the top of the list for women while prostate and lung account for the vast majority in males. These numbers are expected to double by 2030, mainly because of the increased age of a growing population. The majority of patients are diagnosed late, diagnosis is largely inaccurate, and survival rates are much lower than in any other continent. Malaria, tuberculosis, amoebiasis, typhoid and brucellosis are often diagnosed at the expense of other conditions, including cancer, until it is too late.

Most cancers are treated by herbalists and charlatans masquerading as ‘professors’ of one discipline or another. The few available experts engage in individual, unregulated practices. Costly and toxic curative treatments are offered to terminal patients, while half-hearted treatments are offered to those requiring intensive, curative approaches.

The lack of diagnostic personnel and equipment hampers conclusive diagnosis. National cancer control programmes are mainly nonexistent and political expediency drives all policy. Even well-trained personnel resort to outdated shortcuts as a ‘something is better than nothing’ mentality prevails.

Inequitable distribution of facilities and services leave the great majority of the population to their own fates. Many cancers have known modifiable risk factors, if not causes, yet the will to face the challenge for their control is lacking.

A number of solutions were proposed, which included the improvement of economies, accelerated training of pathology personnel, enhancement of cancer diagnosis and treatment infrastructure, and intensification of early detection and prevention strategies, amongst the others.

### A pilot curriculum for the implementation of 3D-CRT

Dr Oniyinye D Balogun talked about radiation therapy centers in developing countries that are beginning to adopt 3D-CRT. Standardised instruments for assisting RT professionals as they transition from 2D to 3D-CRT planning are lacking. Funded by a peer-reviewed departmental grant, we pilot-tested a 2-week curriculum for implementing 3D-CRT for breast cancer, designed to provide the basic foundations for RT professionals. An additional aim of the project was to assess feasibility to use this as a model for wider application in other centers.

The presenter described a pilot site, the National Center of Oncology in Yerevan, Armenia, which acquired a CT simulator and linear accelerator in 2014. The training curriculum for radiation oncologists (ROs), therapists (RTTs) and medical physicists (MPs) of this pilot study consisted of two intensive weeks, which included, inter alia, lectures on the differences between 2D & 3D-CRT and 3D-CRT benefits, lectures on prone positioning, review of the RTOG Breast Cancer Atlas, practical training in supine and prone CT simulation, and feedback from participants using focus groups and questionnaires.

Ten RT professionals participated in the pilot curriculum (six ROs, two MPs and two RTTs) and the assessment demonstrated that contouring was widely considered the most useful aspect of the curriculum. The key challenges included achieving individual participation in all aspects of the curriculum in a high-volume department (~100 patients/day with three machines) and limited use of the CT simulator, and hence, CT simulation practice due to the cost associated with its use.

In conclusions, a pilot curriculum to guide 3D-CRT transition is feasible. It requires the availability of a team of RT professionals from both collaborating institutions and sufficient flexibility from all parties. It presents a unique opportunity for an exchange of expertise. Based on the initial success, this intervention has the potential to benefit other developing countries and should be adapted to the needs of each centre.

### Oncology training and capacity building

Training in clinical oncology, building up capacity, translational research and challenges in eastern Africa were discussed by a group of panelist chaired by F Asirwa.

#### Training in traslational research: opportunities and challenges in East Africa

DINO AMADORI, the brain and energy behind the Mwanza project and Bugando medical centre, emphasised that translational research remains today the most relevant field of preclinical and clinical research in oncology. Basic research allows us to understand the fundamental aspects of phenomena (cancer disease in our interest). This knowledge then has to be used (through clinical trials) to produce new drugs, devices and treatment options for patients.

Precision medicine includes treatments targeted to the needs of individual patients on the basis of genetics, biomarkers, and phenotypic or psychosocial characteristics during all stages of care, including prevention, diagnosis, treatment and follow-up. The goal is to improve clinical outcomes for individual patients by minimising unnecessary side effects. Individual patients have specific needs, which are the result of their genetic makeup and exposure to environmental risk factors. Health systems have to provide diagnostics, informatics and decision-making support to health care providers.

As more progress is made each day in oncology, a personalised treatment with the tailoring of cancer treatments to individual characteristics will be the next step.

#### The role of biobanking in cancer research

One of the last presentations highlighted the role of biobanking in joint African collaborations, and was given by Maimuna Mendy from IARC. This topic was followed by the role of government and ministries of health in advocacy and maintaining the quality of pathology services.

The biobank presentation introduced key principles of biobanking, its relevance in cancer research and ethics, legal and issues (ELSI) that should be taking into account from recruitment, sample acquisition, storage use and re-use of samples. A biobank is defined as a collection of biological material and their associated data, and it is rapidly being recognised as an important infrastructure for cancer research. As medicine evolves, researchers are identifying increasing numbers of molecular analytes and biomarkers that characterise both healthy and medical conditions.

However, due to limited resources and related constraints, biobank introduction in African health service institutions is occurring at a slower pace compared to non-African countries [4]. In addition, many African countries are yet to develop standard guidelines and protocols regulating the collection, processing, storing and sharing of biological samples for future unspecified use. The established procedures and protocols we used in Africa should be in line with international guidelines, while also taking into account national and local norms.

The importance of biobanking in prospective studies and linkage to cancer registries as a research platform and to monitor cancer incidence, has been demonstrated in the example of the Gambia Hepatitis Intervention study-GHIS [5] The GHIS was initiated in 1992 with the aim of determining the efficacy of HBV vaccination to prevent infection and liver cancer. At the same time, the national cancer registry was established in close collaboration with pathology services to record and monitor cancer incidence, thus providing a reliable platform for documenting the endpoint for the intervention study. The GHIS benefited from the well-established biobank in Gambia that stored, managed and provided the valuable biological samples and data for the vaccine efficacy and follow-up studies [6].

With the cancer burden projected to increase by 70 % over the next two decades in LMICs, including Africa, and as the complexity of diseases also increases, largely due to multifactorial risk factors including environmental and genetic factors, it is the right time for African institutions to establish infrastructures to ensure the availability of African biological resources for multidisciplinary research in order to contribute to cancer control and public health.

#### Summary of the meeting and the plan of action

A number of solutions were proposed, which included along the improvement of economies, accelerated training of pathology personnel, enhancement of cancer diagnosis and treatment infrastructure and intensification of early detection and prevention strategies. Other more innovative ideas included a case for a global cancer fund, telepathology and a consideration for an African pathology school.

Expanding the success of the Bugando model could be translated in sharing its lessons more widely through scientific presentations and hosting further training session.

The participants have agreed to set up four committees with the mandate to develop in short term the action plan in the domain of Clinical oncology, Pathology, Telemedicine/Telepathology and Research. The committees will work in collaboration with the existing committees in AORTIC and plan a follow up meeting next year in Eastern Africa.
